# Transmission of Swine Influenza A Viruses along Pig Value Chains, Cambodia, 2020–2022

**DOI:** 10.3201/eid3012.240695

**Published:** 2024-12

**Authors:** Arata Hidano, Dina Koeut, Hannah Holt, William T.M. Leung, Sokhom Krean, Vutha Chhim, Bunnary Seng, Sovanncheypo Chao, Wong Foong Ying, Pov Son, Sina Vor, Sokchea Huy, Ty Chhay, Sothyra Tum, San Sorn, Monidarin Chou, Yvonne C.F. Su, Gavin J.D. Smith, James W. Rudge

**Affiliations:** London School of Hygiene and Tropical Medicine Faculty of Public Health and Policy, London, United Kingdom (A. Hidano, H. Holt, W.T.M. Leung, J.W. Rudge); Ministry of Agriculture, Forestry and Fisheries National Animal Health and Production Research Institute, Phnom Penh, Cambodia (D. Koeut, S. Krean, V. Chhim, B. Seng, S. Chao, S. Tum, S. Sorn); Duke – National University of Singapore Medical School, Singapore (W.F. Ying, Y.C.F. Su, G.J.D. Smith); Livestock Development for Community Livelihood Organization, Phnom Penh (P. Son, S. Vor, S. Huy, T. Chhay); University of Health Sciences, Phnom Penh (M. Chou)

**Keywords:** influenza, influenza A, viruses, swine flu, zoonoses, swine, epidemiology, surveillance, low- and middle-income countries, One Health, farms, slaughterhouses, abbatoirs, Cambodia

## Abstract

We analyzed >4,000 pig samples from slaughterhouses in Cambodia and found higher influenza A seroprevalence (40.0%) and prevalence (1.5%) among pigs from commercial farms than smallholder farms (seroprevalence 8.9%; prevalence 0.6%). Multivariable analyses revealed evidence of transmission after leaving farms. Findings have implications for influenza risk and surveillance in emerging livestock systems.

Swine influenza A viruses (IAVs) contribute to risk for pandemic emergence in humans. Emerging livestock systems in low- and middle-income countries (LMICs) have been proposed as hotspots for novel viruses because of the proximity between avian, swine, and human host populations, high densities of smallholder and multispecies farming systems with poor biosecurity, and rapid growth in livestock industries (*1*–*3*). However, systematic surveillance of swine IAVs in those settings is nearly nonexistent, limiting our understanding of IAV epidemiology and evolution. We conducted slaughterhouse sampling of pigs over a 2-year period in Cambodia to compare IAV circulation in smallholder versus commercial farms and identify risk factors associated with active IAV infection at slaughterhouses. By performing IAV surveillance in slaughterhouses, we assessed the role of transmission during transport and at slaughterhouses and examined implications for epidemiologic inference of IAV risk along pig value chains, the series of interconnected activities encompassing the production, distribution, and processing of pigs. 

## The Study

We selected 18 slaughterhouses in 4 provinces in Cambodia to encompass pigs from smallholder (<100 pigs) and commercial farms (>100 pigs), after conducting a rapid assessment survey among 52 slaughterhouses to characterize their operations (Appendix). We tested pigs monthly at each slaughterhouse during March 2020–July 2022 (*4*). We based sample sizes for each batch (i.e., pigs from the same source tested on the same day at a given slaughterhouse) on 95% probability of detecting >1 positive animal if prevalence within an infected batch was >20% (*5*). We extracted RNA from nasal swab samples and screened for active IAV shedding using real-time RT-PCR targeting the IAV M gene (*6*). We screened blood serum samples for IAV nucleoprotein antibodies using ID Screen Influenza A Multi-species ELISA (Innovative Diagnostics, https://www.innovative-diagnostics.com). We collected data on pig breed, age, type, and origin during each sampling visit. 

Our study was approved by ethics committees at the London School of Hygiene and Tropical Medicine Institutional Review Board (approval no. 16635) and the Animal Welfare and Ethical Research Board (reference no. 2019-12), National Ethics Committee for Health Research in Cambodia (reference no. 105), Human Research Protection Office (reference no. A-21055), and Animal Care and Use Review Office of the US Army Medical Research and Development Command Office of Research Protections.

To account for chronological and other directional relationships between variables, we developed a directed acyclic graph assuming IAV antibodies are detectable >7 days after exposure (*7*). ELISA-determined serostatus likely represented IAV exposure on farms because pigs stayed at slaughterhouses only <6 days in this study; virus shedding by pigs starts as early as 1 day after IAV infection and can last >5 days (*7*). Thus, positive PCR results (i.e., positive infection status) might indicate IAV exposure on the farm shortly before departure to a slaughterhouse, during transport, or at the slaughterhouse. 

We developed Bayesian hierarchical logistic regression models to estimate the direct effect of each exposure, adjusted for confounding and batch-clustering effects. We used batch size and duration of stay at a slaughterhouse as continuous variables using fractional polynomial and generalized additive models and categorical variables. We selected functional forms with the largest Bayes factors. We estimated posterior adjusted odds ratios (aOR) using Stan version 2.26.1 (*8*). We explored spatial trends in seroprevalence based on location of batch origin. We conducted a sensitivity analysis to quantify the potential effects of imperfect diagnostic tests (Appendix). 

We sampled 616 batches from 18 slaughterhouses, which provided 4,089 swab and 4,069 serum samples; 340 (55.2%) batches were from commercial and 204 (33.1%) were from smallholder farms in Cambodia, 59 (9.6%) batches were imported from Thailand, and 13 batches were of unknown origin. Estimated transport durations within Cambodia were 0.1–10.1 hours. At slaughterhouses, pigs were penned in groups of 3–30 and kept an average of 3–36 hours before slaughter, depending on the slaughterhouse. Most slaughterhouses reported that pigs were kept 1–6 days. Pens were cleaned daily in 15 slaughterhouses, weekly in 2, and monthly in 1. At least 1 pig tested positive for active infection in 37 (6.0%) batches and for seroconversion in 355 (59.1%) batches (Table). 

**Table:** Batch- and slaughterhouse-level results from pig sampling, stratified by slaughterhouse province in a study of transmission of swine influenza A viruses along pig value chains, Cambodia, 2020–2022*

Characteristics	Overall	Slaughtherhouse province			
		Kampong Speu	Kandal	Takeo	Phnom Penh
Slaughterhouses	18	5	6	4	3
Batches	616	200	136	175	105
From commercial farms	397 (64.4)	137 (68.5)	97 (71.3)	94 (53.7)	71 (67.6)
PCR-positive	37 (6.0)	1 (0.5)	15 (11.0)	12 (6.9)	9 (8.6)
ELISA-positive	355 (59.1)	127 (63.5)	75 (55.1)	95 (54.3)	58 (55.2)
Batch size, median (range)	6 (1–120)	5 (1–110)	5 (1–32)	6 (1–31)	20 (2–120)
Samples per batch, median (range)	6 (1–16)	5 (1–16)	5 (1–15)	6 (1–13)	12 (2–16)
Within-batch prevalence, median (range)†	20 (6.7–100)	50	33.3 (6.7–100)	14.3 (10–66.7)	12.5 (7.1–55.6)
Within-batch seroprevalence, median (range)‡	50 (6.7–100)	50 (6.7–100)	58.3 (9.1–100)	50 (10–100)	50 (6.7–100)
Male percentage per batch, median (range)	50 (0–100)	42.9 (0–100)	50 (0–100)	50 (20–100)	50 (0–100)
Finisher percentage per batch, median (range)	100 (0–100)	100 (0–100)	100 (100–100)	100 (0–100)	100 (100–100)
Batches by cleaning frequency of slaughterhouse					
Daily	536 (87.0)	176 (88)	111 (84.1)	175 (100)	74 (70.5)
Weekly	45 (7.3)	24 (12)	21 (15.4)	0	0
Monthly	31 (5.0)	0	0	0	31 (29.5)
Transport duration, h, median (range)	0.8 (0.1–10.1)	0.5 (0.2–7.9)	1.5 (0.5–9.9)	0.3 (0.1–10.0)	2.1 (0.9–10.1)
Duration at slaughterhouse, h, median (range)	12 (2–144)	10 (2–48)	12 (5–48)	12 (5–144)	8 (5–20)
Batches by location of originating farm					
Kampong Speu Province	329 (53.4)	177 (88.5)	69 (52.3%)	39 (22.3)	44 (41.9)
Takeo Province	133 (21.6)	2 (1.0)	0 (0)	125 (71.4)	6 (5.7)
Kampong Chhnang Province	42 (6.8)	14 (7.0)	0 (0)	0 (0)	28 (26.7)
Cambodia, other province	53 (8.6)	6 (3.0)	36 (27.3)	3 (1.7)	4 (3.8)
Imported from Thailand	59 (9.6)	1 (0.5)	27 (20.5)	8 (4.6)	23 (21.9)

*Values are no. (%) except as indicated. †PCR confirmed. Within-batch prevalence and seroprevalence were calculated among positive batches (i.e., with >1 positive pig). Range is not provided for Kampong Speu for within-batch prevalence because only a single batch tested PCR-positive. ‡ELISA confirmed.

**Figure 1:**
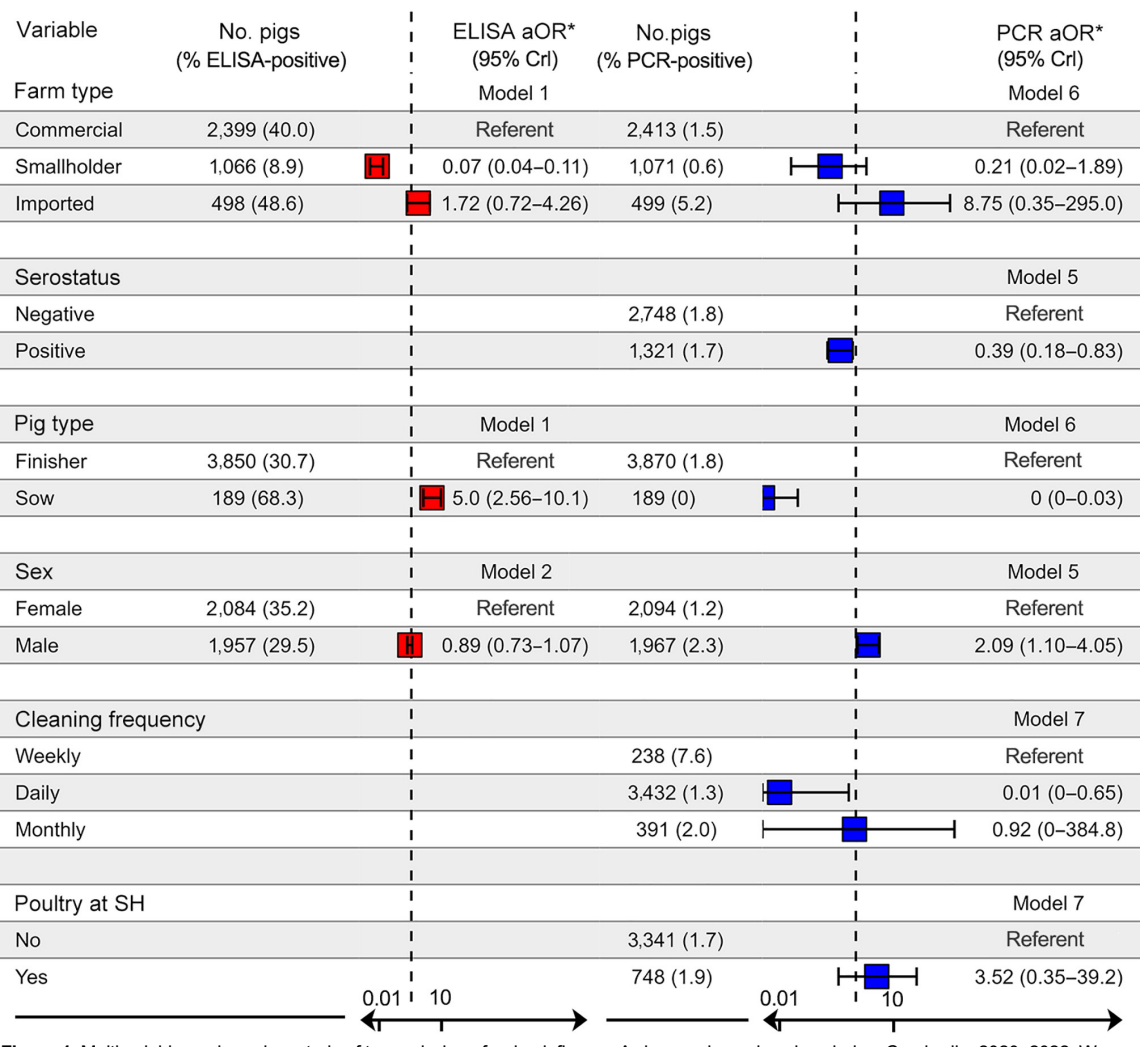
Multivariable analyses in a study of transmission of swine influenza A viruses along pig value chains, Cambodia, 2020–2022. We analyzed exposure variables for associations with ELISA-confirmed influenza A serostatus (red) and PCR-confirmed active infection (blue) at the individual-pig level. Boxes indicate mean, horizontal bars attached to boxes indicate 95% CrI, vertical dotted lines indicate aOR = 1. We estimated posterior aORs and 95% CrI, shown on a log scale, using Bayesian hierarchical regression models derived from a directed acyclic graph. *Model numbers indicated in aOR columns correspond to models described in Appendix Table 1. 95% CrI, 95% credible interval; aOR, adjusted odds ratios; SH, slaughterhouse.

**Figure 2:**
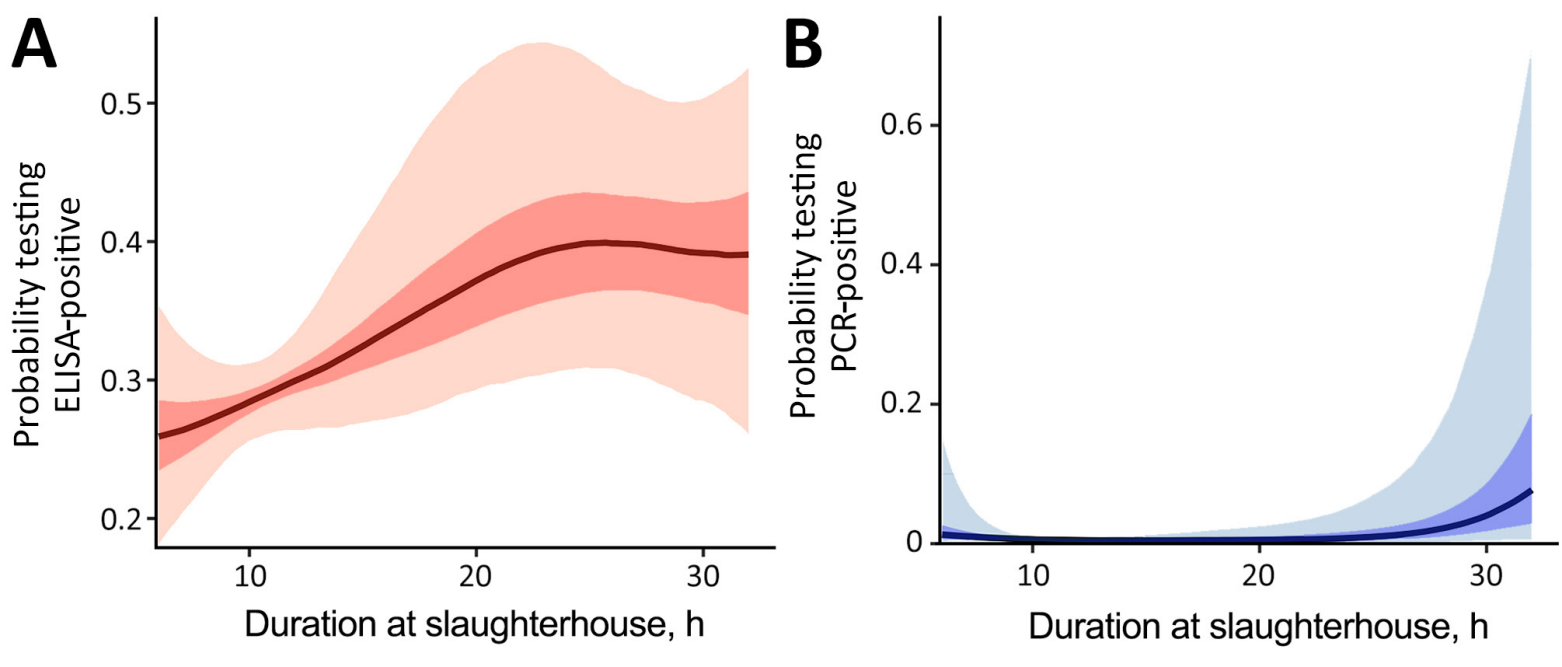
The adjusted probability of testing positive in a study of transmission of swine influenza A viruses along pig value chains, Cambodia, 2020–2022. A) Probability of ELISA-positive; B) probability of PCR-positive. Adjustments are a function of the duration at slaughterhouses, but other variables are kept at baseline. Solid lines indicate predicted means; dark shading indicates 50% CrI and light shading, 95% CrI.

**Figure 3:**
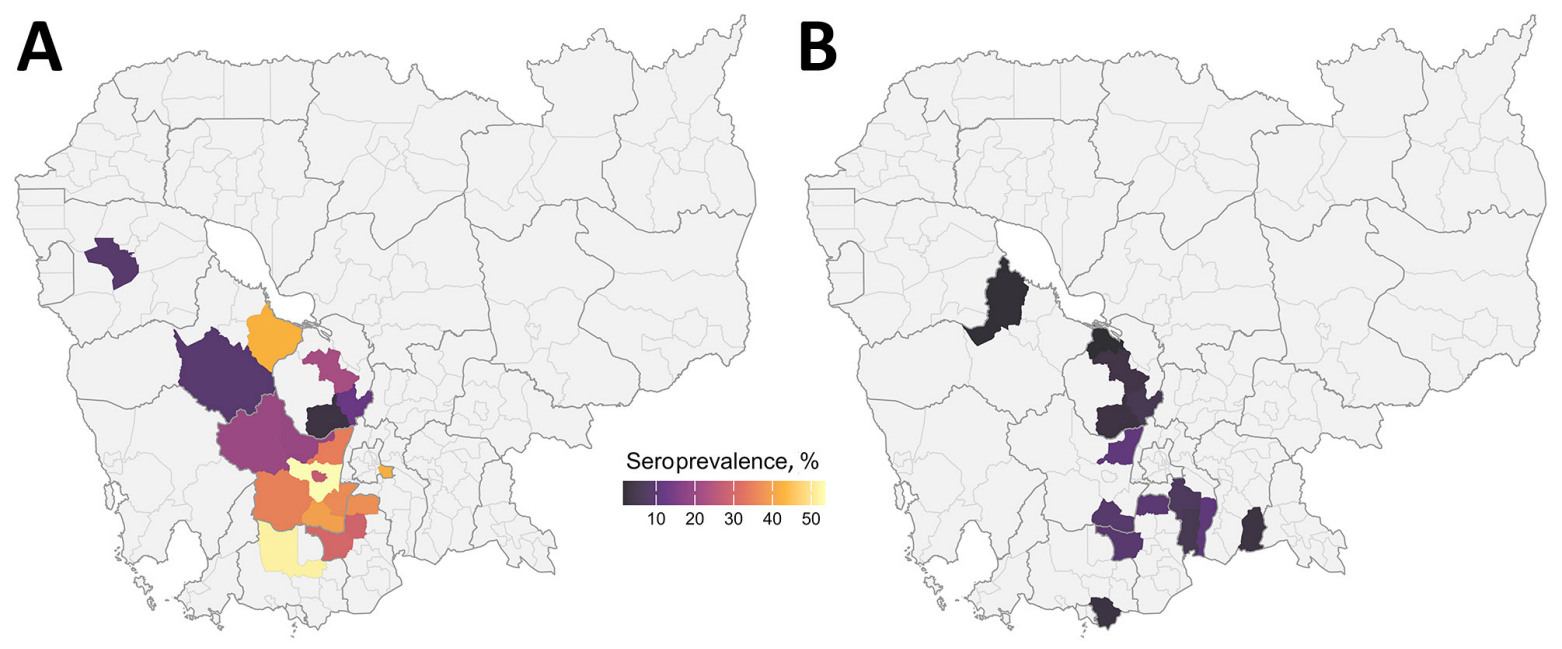
Spatial distributions of adjusted seroprevalence in a study of transmission of swine influenza A viruses along pig value chains, Cambodia, 2020–2022. Distribution by district of origin among commercial farms (A) and small-scale farms (B). Average seroprevalence was estimated for districts that had >2 batches of pigs from the same source sampled on the same day at a given slaughterhouse.

Seroprevalence among commercial farm pigs was 40.0%, considerably higher than among pigs from smallholders (8.9%). In multivariable analyses, pigs from smallholders were less likely to test seropositive (aOR 0.07; 95% credible interval [CrI] 0.04–0.11) than pigs from commercial farms. Infection prevalence was also lower among smallholder (0.6%) than commercial farm pigs (1.5%), although that association was not statistically significant after adjusting for confounders (Figure 1). Odds of active infection were lower among seropositive pigs (aOR 0.39; 95% CrI 0.18–0.83) and among sows. Several associations provided evidence of transmission at slaughterhouses; specifically, active infection was substantially lower among pigs sampled at slaughterhouses that cleaned pens daily compared with slaughterhouses that cleaned weekly, and increased with duration at the slaughterhouse. We also noted a positive trend between a longer stay at slaughterhouses and seroprevalence (Figure 2, panel A), possibly reflecting risk for exposure shortly before or during transport to the slaughterhouse. The presence of poultry at slaughterhouses did not affect active infection status. Associations were not substantially affected by potential underdetection of infection in a sensitivity analysis (Appendix Table 4). For commercial but not smallholder farms, seroprevalence averaged across batches varied among districts (Figure 3). 

## Conclusions 

Our findings demonstrate higher IAV circulation among pigs from commercial than from smallholder farms, adding information to limited studies on swine IAV epidemiology in LMICs. The seroprevalence at commercial farms in Cambodia was comparable to that in high-income countries (*9*). The large variation in seroprevalence among batches from commercial farms, even farms owned by the same company, might reflect spatiotemporal variation in transmission, but warrants further investigation of the contribution of farm management practices. Literature provides evidence of IAV persistence and evolution through successive reassortments on commercial farms (*10*). Our findings highlight how increased livestock population and density in LMICs might increase risk for novel IAV emergence and amplification. As reported elsewhere, phylogenetic inferences from our samples from Cambodia identified 9 distinct swine IAV lineages, with human H1N1/pdm09 virus lineages predominating (*4*). The novel European avian-like H1N2 reassortant variant, possessing G4-like H1 sequences, was also present in 2 batches. Those batches, which we sampled within 24 hours of shipment, originated from different commercial farms at different timepoints, indicating the potential spread of this novel swine IAV variant among commercial farms in Cambodia.

Although little is known about IAV transmission during transport and at slaughterhouses (*11*), our results indicate traders and slaughterhouse workers might be at heightened risk for swine IAV exposure. We are currently developing novel microbead-based serologic assays to distinguish antibodies to different IAV subtypes among pigs and humans, which will augment our understanding of IAV dynamics within and between different farm types and host species. In addition, reduced time from farm to slaughterhouse, less stressful pig handling, and improved slaughterhouse hygiene may ameliorate both enzootic and zoonotic transmission risks during the final stages of the pig value chain. 

In summary, our analyses indicate that active infections among pigs sampled at slaughterhouses might reflect exposure immediately before or during transport to or at slaughterhouses. Thus, slaughterhouse surveillance data should be interpreted with caution when inferring risk from farm types or geographic origin, even when data on pig origin are available. In LMICs, surveillance at slaughterhouses rather than farms may be the only sustainable option (*12*). That surveillance should be coupled with monitoring of the status of pig value chains, which can change rapidly because of pig sector growth and outbreaks of diseases, such as African swine fever. Those findings contain implications for influenza risk and surveillance in emerging livestock systems. 

Appendix Additional information on transmission of swine influenza A viruses along pig value chains, Cambodia, 2020–2022.
