# Supplementary material for: Transmission of Swine Influenza A Viruses along Pig Value Chains, Cambodia, 2020–2022
Source: Emerg Infect Dis. 2024 Dec;30(12):2669–73. doi: 10.3201/eid3012.240695 (PMC11616665; doi:10.3201/eid3012.240695)
Supplement: Appendix — Additional information on transmission of swine influenza A viruses along pig value chains, Cambodia, 2020–2022. [file 24-0695-Techapp-s1.pdf]

Article DOI: https://doi.org/10.3201/eid3012.240695

EID cannot ensure accessibility for supplementary materials supplied by authors. Readers who have difficulty accessing supplementary content should contact the authors for assistance.

Transmission of Swine Influenza A Viruses along Pig Value Chains, Cambodia, 2020–2022

Appendix

Materials and Methods

Rapid Slaughterhouse Assessment

A total of 52 registered slaughterhouses (SHs) were visited in 4 provinces in 2019 to understand SH characteristics in the study provinces. These provinces were primarily selected due to the diversity of pig farming systems (e.g., commercial farms and smallholders) present in this region of Cambodia, along with our prior experience working with the pig sector in this region and logistical feasibility due to proximity to Phnom Penh. Of those visited, 9 were in Takeo Province, 15 in Kandal, 15 in Phnom Penh and 13 in Kampong Speu. All those interviewed accepted the Department of Animal Health visiting the SH to sample the pigs. All SHs reported operating throughout the year, and all except 4 reported operating 7 days per week. In terms of throughput, the median, maximum and usual number of pigs slaughtered at the SH per day was 32.5 (range: 10–300) and 9.5 (1–200), respectively. Many SHs received pigs in the afternoon and killed the pigs in the very early hours of the morning. The median total area of SH and area of slaughtering facilities was 1,470 m^2 (range: 200–10,000) and 300 m^2 (range: 35–2,520), respectively. All the SHs were privately owned. SH owners have to ‘bid’ to the government to be one of the operating SHs every 5 years. Therefore, it is hard to invest in improvements in SHs as the site might change location after a few years. This is reflected in the results of this assessment where the median time SH had been in operation was 4 years (range 1–4 years).

Those slaughtering sows (cull sows) were mostly in Takeo or Kampong Speu provinces. In 5 of these SHs cull sows comprised 5% or less of pigs slaughtered, 3 estimated that cull sows comprised 10% of pigs slaughtered and 4 estimated they comprised 20% or more (max 50%) of pigs slaughtered. All SHs that reported slaughtering exotic breeds were in Kampong Speu. Pigs came mostly from Cambodia with some SHs sourcing pigs from Thailand. Before the outbreak of ASF, 4 SHs had received pigs from Vietnam. Many pigs were sourced from Kampong Speu, Kandal, and Takeo. Two SHs estimated that they got 15% of their pigs from a mix of Pursat, Battambang, Siem Reap and Kampong Thom without breaking this down further. Around half of the SHs said some of the pigs came from smallholder households. When investigating this by province, Kampoung Speu (9 out of 11), Takeo (7 out of 9) and Kandal (8 out of 15) SHs mentioned being supplied by smallholders more than Phnom Penh (1 out of 15). This assessment ensured that the provisional sampling strategy (see below) could achieve the required sample size in a given study period and sample pigs from diverse origins. The assessment also informed the questionnaire design.

Sampling

Two districts per province were selected randomly with a probability proportional to the pig population size. All SHs in selected districts were recruited in the study. If no SH existed in a selected district, the nearest SH in a neighbouring district was chosen. Sampling was piloted in early March 2020 to ensure all planned biosecurity procedures were feasible and followed. Sampling was conducted by staff of the National Animal Health and Production Research Institute, Cambodia and Livestock Development for Community Livelihood Organization between March 2020 and July 2022 with some interruptions during this period due to SARS-CoV-2 measures in Cambodia. No sampling was conducted between April 2020 to May 2020 and April 2021 to May 2021. Each participating SH was visited once a month with several exceptions: visits to SHs in Kandal were not made in September 2020 and June 2021; SH No.9 suspended its operations between March 2020 and January 2021, and sampling was not conducted; SH No.10 terminated its operations in December 2020 and no sampling was conducted thereafter.

The assumed 20% within-herd prevalence was set by slightly inflating the seroprevalence of H1 reported by (*1*) to account for infection with other subtypes. To provide a reference of the

range of sample sizes per batch, all pigs within the batch were sampled if the batch size was 9 or less, and up to 16 pigs randomly selected pigs were sampled if the batch size was 101 or above.

Pigs were restrained using snares and swab samples collected from both nostrils using a circular rotating motion, which were transferred into a viral transport medium and kept at 4 °C until arrival at the laboratory. A maximum 5ml of blood was collected each into EDTA tube and non-EDTA tube. During each sampling visit, relevant stakeholders (either veterinarians, butchers, traders, or SH owners) were interviewed to obtain the following batch- and pig-level information; batch size, batch origin, vaccination status, age, sex, breed, type (finisher, sow or other), body condition score and any clinical signs. At the NAHPRI laboratory nasal swabs were stored at –80 °C, and serum samples were separated within 48 hours of collection. Swab samples from the same batch were pooled by combining up to 5 samples. Individual PCR status was then determined for pools that tested positive.

Statistical Analysis

The distance and transport time between the origin and the SH were calculated for batches with known origin using the R package gmapsdistance v4.0.4 (*2*), which computed the distance and time between the centroid of the origin district and the SH location using the Google Maps Distance Matrix API. The transportation mode was set to 'driving', the departure time set to 5pm, and the traffic model set to 'None'. All imported batches had transport time greater than 7 hours, whereas all Cambodian batches had transport time shorter than 5 hours. We therefore categorized the transport time variable to estimate the effect of import and transport time. Given the 25th and 75th percentiles for the transport time among Cambodian batches were 0.80 and 1.97 hours, we used three levels for this variable; less than 1 hour, equal to or greater than 1 hour and less than 2 hours; equal to or greater than 2 hours. We used the duration that each SH reported to keep pigs on average as the proximal duration that batches stayed at a given SH. The effect of the presence of a specific type of animal was estimated using a separate model for each of poultry in SH, poultry in neighbouring houses, dogs in SH, cats in SH, and slaughtering cattle. No other animals were identified.

A direct acyclic graph (DAG) was developed to depict the causal relationship between variables using Dagitty R-package (*3*) (Appendix Figure 1). A Cauchy distribution (location = 0, scale = 10) was used as a weakly informative prior distribution for all exposure variables and the

default prior (student $t(3,0,2,5)$) was used for all intercepts. The posterior odds ratios of the exposure variables were estimated through the Hamilton Monte Carlo (HMC) method using Stan v2.26.1 through brms package v2.20.1 (*4*) by running three independent chains for at least 4000 iterations each and discarding the first 1000 samples as burn-in. The model convergence was confirmed by checking Rhat values to be <1.01, large effective sample sizes, and trace plots for good mixing (Appendix Figure 2). We modelled the following variables using specific form and their fits were compared based on Bayes factor using bayestestR package v0.13.1 (*5*): duration of stay at SH using fractional polynomial (FP) or, generalised additive models (GAM); batch size using FP, GAM, or a categorical variable. For the FP model, we identified the best functional forms of variables by setting the degrees of freedom to be 4 and alpha-level 0.25 using mfp package v1.5.4 (*6*). Bayes factor >3 was considered strong evidence against the null hypothesis and <0.3 to be strong evidence for the null hypothesis; the model with the highest Bayes factor was chosen (Appendix Table 1).

Sensitivity Analysis

While non-differential misclassification in which the diagnostic outcome is independent of exposures of interest biases the estimate towards null, differential misclassification can bias the estimate in both directions (*7*). We therefore evaluated the impact of various misclassification scenarios on the outcome. Accuracy estimates for IAV PCR using pig nasal swabs are scarce in literature, but one study assumed the diagnostic sensitivity and specificity to be 90% and 100%, respectively (*8*). Pigs at smallholders are kept in open pens, hence likely to get infected through direct contact rather than aerosol; pigs in commercial farms also get infected through aerosol (*9*,*10*). Literature suggests that viral shedding is less when infected through contact than through aerosol (*11*). Therefore, pigs at smallholders may shed less virus and the PCR sensitivity may be lower for these pigs. We carried out the sensitivity analysis by assuming that the PCR sensitivity is low at 40% for smallholder pigs, 100% specificity, and 2% true shedding prevalence, we imputed 'true' outcomes and created 50 new datasets, which were fitted to the final model to estimate each parameter of interest.

Results

Slaughterhouse Characteristics

There was a large within- and between-slaughterhouse variation in terms of pig slaughtering activities among the 18 SHs. The smallest SH processed, one average, 2 pigs per day, and the largest processed 90 pigs per day; across the 18 SHs, median throughput was 8 pigs per day. In terms of within-SH variation, one SH did not process any pigs on some days, and the largest SH processed up to 120 pigs per day. Except for one SH that kept each pig in separate pens in which pigs could not contact each other, all SHs grouped pigs from the same origin in the same pen. However, 13 SHs reported pigs from different origins may contact each other. The minimum and maximum number of pigs kept in a pen was 3 and 30 (median: 8). The average duration that pigs stayed at each SH ranged between 6 and 32 hours and the longest duration ranged from 10 hours to 6 days. Three SHs slaughtered cattle. Several SHs also reported the presence of poultry (3 SHs), dogs (9), and cats (1) on their premises. Out of 9 SHs with dogs, 7 SHs reported contact between these dogs and pigs. Cats in 1 SH could also contact pigs. Three SHs reported that their neighbors had backyard poultry, which could contact SH pigs. We observed that wild or domesticated birds could access pig holding areas at all the SHs.

Pig Batch Characteristics

The median number of pigs per batch was significantly larger (Wilcoxon rank-sum test, $p < 0.001$) for commercial farms (median = 9; Q1 = 6, Q3 = 22) than for smallholders (median = 7; Q1 = 5, Q3 = 13). The median number of pigs sampled was 5 for batches from smallholders (range 1–14) and 7 for those from commercial farms (range 1–16). When stratified by origin province, 330 batches (53.7%) were from Kampong Speu Province, followed by Takeo 133 batches), Thailand (59 batches), and Kampong Chhnang (42 batches). For 604 batches, we calculated the distances and transportation duration between the origin district and SH. Estimated transport distances (and corresponding durations) within Cambodia ranged from 2.36km (0.1 hours), for movement within Takeo, and up to 579km 10.1 hours) for movement of imported pigs from the Cambodia-Thailand border and Takeo. While the SHs in Phnom Penh frequently received pigs from Thailand until July 2021, those in Kampong Speu and Takeo often sourced pigs locally (Appendix Figure 3). The median distances that batches moved to each province were 19.3km, 65.5km, 80.5km, and 17.4km for Kampong Speu, Kandal, Phnom Penh,

and Takeo, respectively. The median durations required for transport were 0.5, 1.47, 2.11, and 0.33 hours, respectively. Batches from smallholders moved significantly shorter distances than those from commercial farms after controlling for the slaughterhouse province (linear regression coefficient = –86.2km, $p < 0.001$).

Modelling the PCR and ELISA outcome

Different sets of variables were included in the regression model to account for confounding effects for each exposure of interest (Appendix Table 2). Two variables (duration at SH and batch size) were modelled as continuous using fractional polynomial or generalized additive models, or categorical variables. The best functional form was chosen based on Bayes factor for each model that included the outcome and exposure of interest (Appendix Table 1).

In addition to the results presented in the main manuscript, batch size did not have any clear effect on the ELISA outcome (Appendix Figure 4), yet batches with 21–30 pigs were less likely to test PCR positive compared to batches with 1–10 pigs (Appendix Table 3). Transport duration did not have a clear effect on both the ELISA and PCR outcomes (Appendix Table 3). The adjusted odds ratio (aOR) for mixing pigs from different origins at SH on the PCR outcome was 0.12 (95% CI 0–12.2). The ELISA and PCR outcomes were not different between years. Presence of poultry, dogs or cats at the SH did not affect the PCR outcome. Pigs in SHs that also slaughtered cattle had smaller odds of testing PCR positive; most of these slaughterhouses cleaned the premises daily and we reason that this practice improved the SH hygiene condition. More detailed SH information (e.g., SH structure, practices less stressful for animals) should be captured in future studies. We reason that cross-species IAV transmission at SH was minimal for our study and indeed the sequencing of our isolates supported this hypothesis (*12*).

Appendix Table 4 shows the results of the sensitivity analysis. The effect of imperfect PCR sensitivity on pigs from smallholders was minimal except for the variable representing the daily cleaning practice; the aOR 95% CI included 1. Appendix Table 5 shows the association between the active infection status and clinical signs observed. No clinical signs had statistically significant associations with the active infection outcome. While this is consistent with general ideas that pigs manifest limited clinical signs during IAV infections, we also note that there can be a variability between different observers in this study.

The estimated random effect for each batch indicates the extent to which unobserved factors affected the outcome after accounting for all the variables included in the model. Here, unobserved factors refer to variables such as pig management practices on each farm, difference in infectiousness between IAV lineages, transport practices (e.g., whether pigs transited at multiple points, how traders handled pigs), and unobserved SH practices that may affect IAV transmissions. Therefore, analyzing where a large variance exists in the random effect provides a direction for future research. The standard deviation of the batch-level random effect was 3.71 (95% CI 2.61–5.14) and 1.94 (95% CI 1.72–2.18) for the PCR and ELISA outcome, respectively, suggesting a large effect of unobserved batch-specific factors, especially for the PCR outcome of commercial pigs and the ELISA outcome of both commercial farms and smallholders (Appendix Figures 6 and 7). For the variance of random effects on the ELISA positivity, SH explained 2.6% and 12.3% for commercial farms and smallholders, respectively (Appendix Figure 8). The origin district explained 4.4% and 5.7% of the variance of the ELISA positivity for commercial farms and smallholders, respectively, and its spatial distribution indicates the presence of unobserved factors important for the ELISA positivity in the farm level rather than province level (Figure 3). Some SHs had large variances of random effects of both the ELISA and PCR outcomes for smallholders pigs (Appendix Figure 8 and 10), which may be attributed to SH-specific practices (including practices of traders who used these SHs), characteristics of smallholders who sold pigs to these SHs, or both. Although we attempted to collect information on some trading practices, such as whether a batch included pigs from multiple sources, the majority of the respondents did not provide this information. The variance of random effects on the PCR positivity estimated for each batch was most explained by SH; 2.7% and 8.9% of the variance was explained for batches from commercial farms and smallholders, respectively, and unobserved SH-level factors facilitated IAV shedding in some SHs. Districts from which batches came explained only 0.9% and 1.8% of the variance for the random effect of the PCR outcome for commercial farms and smallholders, respectively. There was no clear trend in random effects of the ELISA outcome across sampling month in each district, suggesting that the effect of seasonality on IAV transmissions on farm might be limited. We then explored if there were any associations between the random effect of the PCR outcome and that of the ELISA outcome. No clear association was found between the random effect for ELISA and PCR outcome (Appendix Figure 11), suggesting that unobserved factors that affected

the PCR outcome may be distinct from those affected the ELISA outcome. This further supports our interpretation that the ELISA and PCR outcome from SH sampling was driven by different mechanisms. It is useful to conduct a study that quantifies a seroprevalence and active shedding prevalence, as well as genomically characterizing IAV lineages, at each point of pig value chains. Such studies should capture, where possible, detailed transport conditions (e.g., pig density during transport, time required for loading/unloading) and pig stress level e.g., using cortisol level in pig saliva.

References

1. Mastin A, Alarcon P, Pfeiffer D, Wood J, Williamson S, Brown I, et al. Prevalence and risk factors for swine influenza virus infection in the English pig population. PLoS Curr. 2011;3:RRN1209. PubMed

2. Azuero Melo R, Zarruk D. gmapsdistance: distance and travel time between two points from Google Maps [cited 2018 May 27]. https://cran.r-project.org/web/packages/gmapsdistance/index.html

3. Textor J, van der Zander B, Gilthorpe MS, Liśkiewicz M, Ellison GT. Robust causal inference using directed acyclic graphs: the R package 'dagitty.' Int J Epidemiol. 2016;45:1887–94. PubMed https://academic.oup.com/ije/article/45/6/1887/2907796

4. Bürkner PC. brms: an R package for Bayesian multilevel models using Stan. Stat Softw. 2017;80:1–28. https://www.jstatsoft.org/article/view/v080i01

5. Makowski D, Ben-Shachar MS, Lüdecke D. bayestestR: describing effects and their uncertainty, existence and significance within the Bayesian framework. J Open Source Softw. 2019;4:1541. https://joss.theoj.org/papers/10.21105/joss.01541

6. Heinze G, Ambler G, Benner A. mfp: multivariable fractional polynomials [cited 2018 May 27]. https://cran.r-project.org/web/packages/mfp/index.html

7. Rothman KJ, Greenland S, Lash TL. Modern epidemiology, 3rd edition. Philadelphia: Lippincott Williams & Wilkins; 2008.

8. Garrido-Mantilla J, Alvarez J, Culhane M, Nirmala J, Cano JP, Torremorell M. Comparison of individual, group and environmental sampling strategies to conduct influenza surveillance in pigs. BMC Vet Res. 2019;15:61. PubMed https://doi.org/10.1186/s12917-019-1805-0

9. Neira V, Rabinowitz P, Rendahl A, Paccha B, Gibbs SG, Torremorell M. Characterization of viral load, viability and persistence of influenza A virus in air and on surfaces of swine production facilities. PLoS One. 2016;11:e0146616. PubMed https://doi.org/10.1371/journal.pone.0146616

10. Zhang H, Li X, Ma R, Li X, Zhou Y, Dong H, et al. Airborne spread and infection of a novel swine-origin influenza A (H1N1) virus. Virol J. 2013;10:204. PubMed https://doi.org/10.1186/1743-422X-10-204

11. Hemmink JD, Morgan SB, Aramouni M, Everett H, Salguero FJ, Canini L, et al. Distinct immune responses and virus shedding in pigs following aerosol, intra-nasal and contact infection with pandemic swine influenza A virus, A(H1N1)09. Vet Res. 2016;47:103. PubMed https://doi.org/10.1186/s13567-016-0390-5

12. Zeller MA, Ma J, Wong FY, Tum S, Hidano A, Holt H, et al. The genomic landscape of swine influenza A viruses in Southeast Asia. Proc Natl Acad Sci U S A. 2023;120:e2301926120. PubMed https://doi.org/10.1073/pnas.2301926120

Appendix Table 1. Bayes factors for competing models with different functional forms for each continuous variable

ID*	Outcome	Exposure	Model†	Bayes factor
1	ELISA	Farm type, pig type	s(Duration at SH) + s(Batch size)	1
	ELISA		I((Duration at SH/10)^1) + I((Batch size/10)^1	3.66E-07
2	ELISA	Duration at SH, transport duration, year	s(Duration at SH)	1
	ELISA		I((Duration at SH/10)^1)	4.10E-02
3	ELISA	Batch size	I((Batch size/10)^3) + I((Batch size/10)^3)*log((Batch size/10)))	1
	ELISA		s(Batch size)	4.21E+05
	ELISA		Batch size as categorical	37.34
4	PCR	Serostatus, Sex, Batch size	s(Duration at SH) + s(Batch size)	1
	PCR		I((Duration at SH/10)^1) + I((Batch size/10)^1) + I((Batch size/10)^3)	7.96E-07
	PCR		s(Duration at SH) + Batch size as categorical	1.46E+06
	PCR		I((Duration at SH/10)^1) + s(Batch size)	4.99E+04
	PCR		s(Duration at SH) + I((Batch size/10)^1)+ I((Batch size/10)^3)	2.60E-12
	PCR		I((Duration at SH/10)^1) + Batch size as categorical	8.45E+04
5	PCR	Farm type, Pig type	s(Duration at SH) + Batch size as categorical	1
	PCR		I((Duration at SH/10)^-2) + I((Duration at SH/10)^-2 * log((Duration at SH/10))) + I((Batch size/10)^1) + I((Batch size/10)^3)	3.21E-11
	PCR		s(Duration at SH) + s(Batch size)	20.59
	PCR		s(Duration at SH) + I((Batch size/10)^1) + I((Batch size/10)^3)	3.22E-04
	PCR		I((Duration at SH/10)^-2) + I((Duration at SH/10)^-2 * log((Duration at SH/10))) + s(Batch size)	923.02
	PCR		I((Duration at SH/10)^-2) + I((Duration at SH/10)^-2 * log((Duration at SH/10))) + Batch size as categorical	0.1
6	PCR	Duration at SH, transport duration, year	s(Duration at SH) + s(Batch size)	1
	PCR		I((Duration at SH/10)^-2) + I((Duration at SH/10)^-2 * log((Duration at SH/10))) + I((Batch size/10)^1) + I((Batch size/10)^3)	2.48E-05
	PCR		s(Duration at SH) + Batch size as categorical	2.48
	PCR		I((Duration at SH/10)^-2) + I((Duration at SH/10)^-2 * log((Duration at SH/10))) + Batch size as categorical	0.61

ID*	Outcome	Exposure	Model†	Bayes factor
7	PCR	Cleaning frequency, mixing batch, pen size, presence of other animals (including poultry in SH)	s(Duration at SH)	1
	PCR		I((Duration at SH/10)^-2) + I((Duration at SH/10)^-2 * log((Duration at SH/10)))	5.92E+11

*ID represents the baseline denominator model that bayes factor was calculated against (hence Bayes factor=1). Rows without ID are numerator models which were compared against their denominator models that have the same set of exposure variables as indicated in 'Exposure' column. ID, identification number.
†Functional form of continuous variables. S() and I() indicates GAM and fractional polynomial. Batch size category is shown in Table 1. Functional forms shown in bold are the chosen model.

Appendix Table 2. Models used to estimate the effect of each exposure variable and controlled variables*

ID	Exposure of interest	Out-come	Farm type	Sero status	Pig type	Sex	Batch size	Duration at SH	Transport duration	Mixing batch	Year	Cleaning frequency	Presence of other animals	Pen size
1	Farm type, pig type	ELISA	✓		✓	✓	✓	✓	✓		✓			
2	Sex	ELISA	✓		✓	✓								
3	Duration at SH, transport duration, year	ELISA	✓		✓			✓	✓		✓			
4	Batch size	ELISA	✓		✓		✓							
5	Serostatus, sex, batch size	PCR	✓	✓	✓	✓	✓	✓	✓		✓			
6	Farm type, piping type	PCR	✓	✓	✓	✓	✓	✓	✓	✓		✓	✓	✓
7	Cleaning frequency, mixing batch, pen size, presence of other animals (including poultry in SH)	PCR	✓		✓			✓	✓	✓	✓	✓	✓	✓
8	Duration at SH, transport duration, year	PCR	✓	✓	✓	✓	✓	✓	✓	✓	✓	✓	✓	✓

*✓ indicates variables included in the regression model. ID, identification number.

Appendix Table 3. The effect of transport duration, batch size, mixing batch and year on the IAV status*

	ELISA			PCR		
Variable	**aOR**	**2.5% limit**	**97.5% limit**	**aOR**	**2.5% limit**	**97.5% limit**
Transport duration						
<1h	Ref			Referent		
<2h	0.83	0.51	1.37	0.63	0.04	8.07
>2h	1.01	0.50	2.00	0.71	0.01	30.23
Pen size (heads per pen)						
<5				Referent		
<9				7.91	0.73	127.2
<13				0.26	0	16.4
<31				0.07	0	1.36
Batch size						
<10				Referent		
11–20				0.29	0.02	3.38
21–30				0	0	0.32
31–40				0.66	0.02	22.5
>40				1	0.05	19.5
Mixing batch						
N				Referent		
Y				0.12	0	12.2

Variable	ELISA aOR	ELISA 2.5% limit	ELISA 97.5% limit	PCR aOR	PCR 2.5% limit	PCR 97.5% limit
Year						
2020	Ref			Referent		
2021	1.29	0.82	2.09	0.54	0.07	4.14
2022	0.91	0.53	1.54	0.97	0.10	8.96
Poultry in neighbouring house						
N				Referent		
Y				0.21	0.01	3.09
Dog in SH						
N				Referent		
Y, contact pigs				2.14	0.28	17.5
Y, don’t contact pigs				0.02	0	7.69
Cat in SH						
N				Referent		
Y				0.24	0	8.90
Slaughter cattle in SH						
N				Referent		
Y				0	0	0.18

*aOR, adjusted odds ratio; **SH, slaughterhouse.**

Appendix Table 4. Results of a sensitivity analysis to account for the imperfect PCR sensitivity, estimated based on 50 iterations of imputing ‘true’ outcomes for pigs from smallholders that tested negative for PCR.

Variable	PCR aOR	PCR 2.5% limit	PCR 97.5% limit
Farm type			
Commercial farm	Referent		
Smallholder	0.82	0.72	12.6
Imported	6.42	0.64	79.6
ELISA status			
Negative	Ref		
Positive	0.48	0.23	0.97
Daily cleaning			
No	Referent		
Yes	0.05	0.002	1.17
Mix batch			
N	Referent		
Y	0.34	0.01	9.02
Pen size at SH			
<5	Referent		
<9	0.69	0.01	37.8
<13	3.15	0.58	19.9
<31	0.63	0.04	7.96
Pig type			
Finisher	Referent		
Sow	8.43×10^{-8}	2.13×10^{-27}	0.08
Sex			
F	Referent		
M	1.82	1.04	3.24
Year			
2020	Referent		
2021	0.70	0.17	2.83
2022	0.98	0.20	4.81
Batch size			
≤10	Referent		
11–20	0.44	0.06	2.66
21–30	0	0	0.69
31–40	0.78	0.05	10.8
>40	1.10	0.12	10.2
Poultry in SH			
N	Referent		
Y	0.23	0.03	1.73

*aOR, adjusted odds ratio; SH, slaughterhouse.

Appendix Table 5. Associations between the presence of clinical signs and IAV ELISA or PCR outcomes*

	ELISA (n = 4,069)						PCR (n = 4,089)					
Clinical signs	Sign+	T(+)	%†	T(–)	%‡	P§	Sign+	T(+)	%†	T(–)	%‡	p§
Fever	7	3	0.2	4	0.1	0.85	7	1	1.4	6	0.1	0.28
Cough	94	28	2.1	66	2.4	0.65	94	2	2.8	92	2.3	1
Discharge	607	209	16	398	14.5	0.28	610	11	15	599	15	1
Sneezing	1	0	0	1	0.03	1	1	0	0	1	0	1
Other respiratory symptoms	1	1	0.1	0	0	0.71	1	0	0	1	0	1

*+, positive; –, negative.
†Percentage of pigs with the clinical sign among test positive.
‡Percentage of pigs with the clinical sign among test negative.
§p value of chi-squared test with Yates' continuity correction.

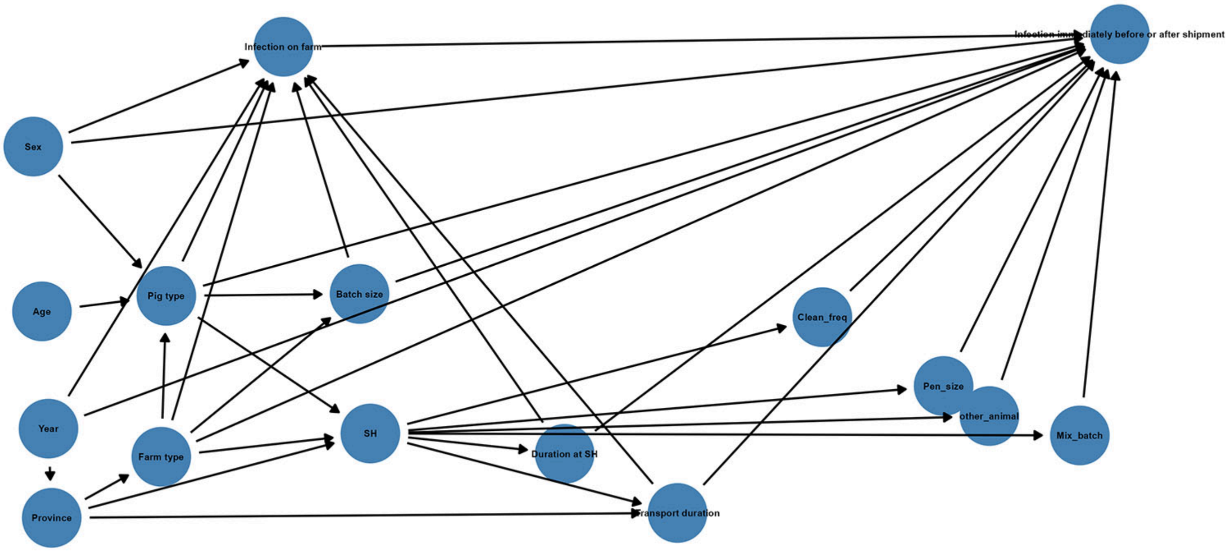

Appendix Figure 1. Assumed directed acyclic graph for the causal relationship between exposures of interest and two outcomes (ELISA and PCR status). Options for variables described in bubbles: Farm type (commercial farms, smallholder farms, or imported), Pig type (sows or finishers), SH (slaughterhouse), Duration at SH (hours pigs stayed at SHs), Transport duration (hours pigs spent in transport), Clean_freq (SH pens cleaned daily, weekly, or monthly), Pen_size (4-level categorical variable representing the number of pigs kept in a pen), Mix_batch (if pigs from different origins kept together in a pen), other_animals (presence of poultry in SH, poultry among neighbors, dogs in SH, cats in SH, or cattle for slaughter in SH), IAV infection on farm (serostatus measured by ELISA), and IAV infection immediately before or after shipment (active infection status measured by PCR). Farm type determined SH because some SH received pigs only from commercial farms or smallholders. Transport duration was determined by the origin province and SH location. SH determined Duration at SH, Clean_freq, Pen_size, other_animals, and Mix_batch as they were all SH-level variables. Farm type affected batch size (larger for commercial farms); batch size was a proxy for herd size and affected the within-herd IAV dynamics. IAV, influenza A virus.

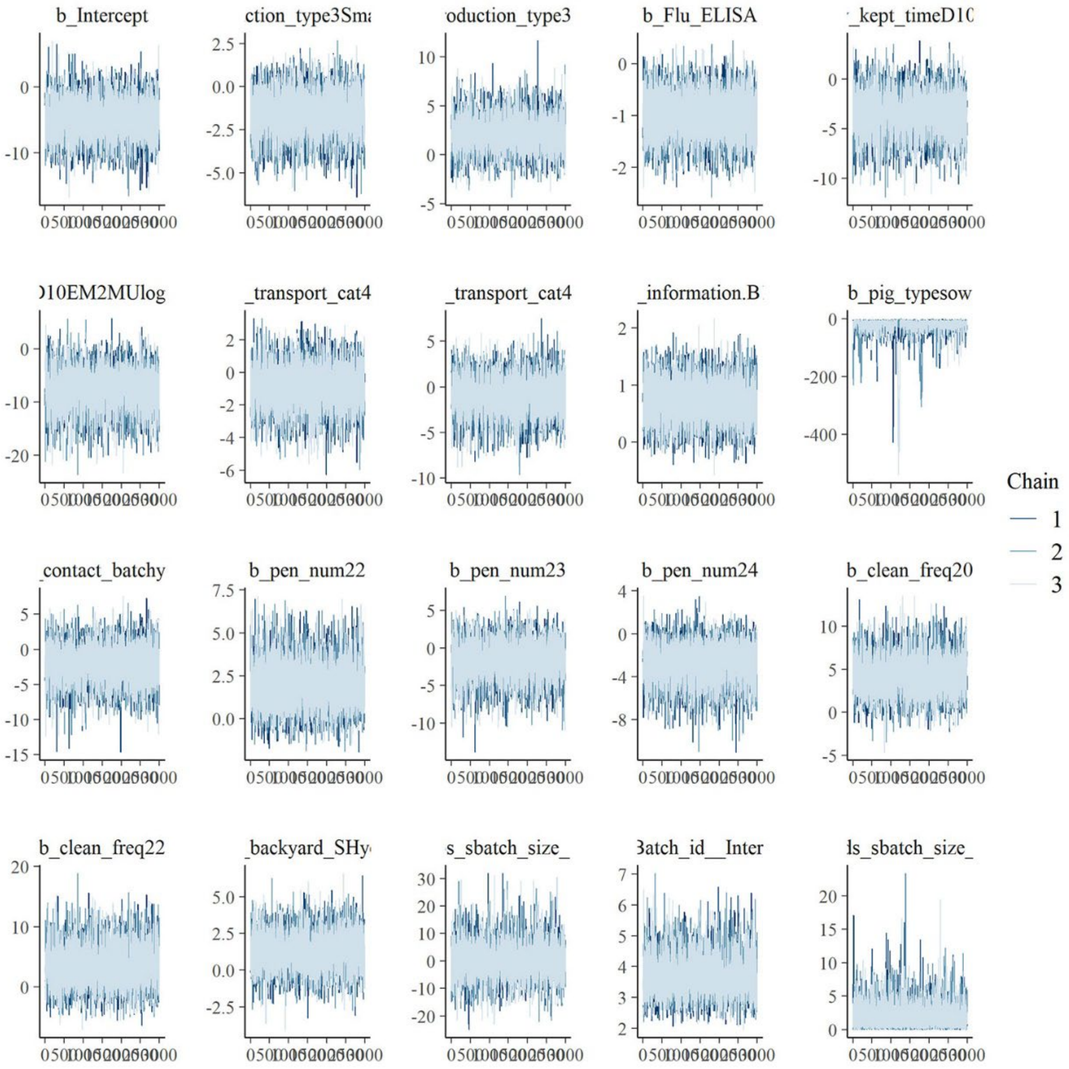

Appendix Figure 2. Trace plots for the Bayesian random effect model for the PCR status with the production type as the exposure of interest.

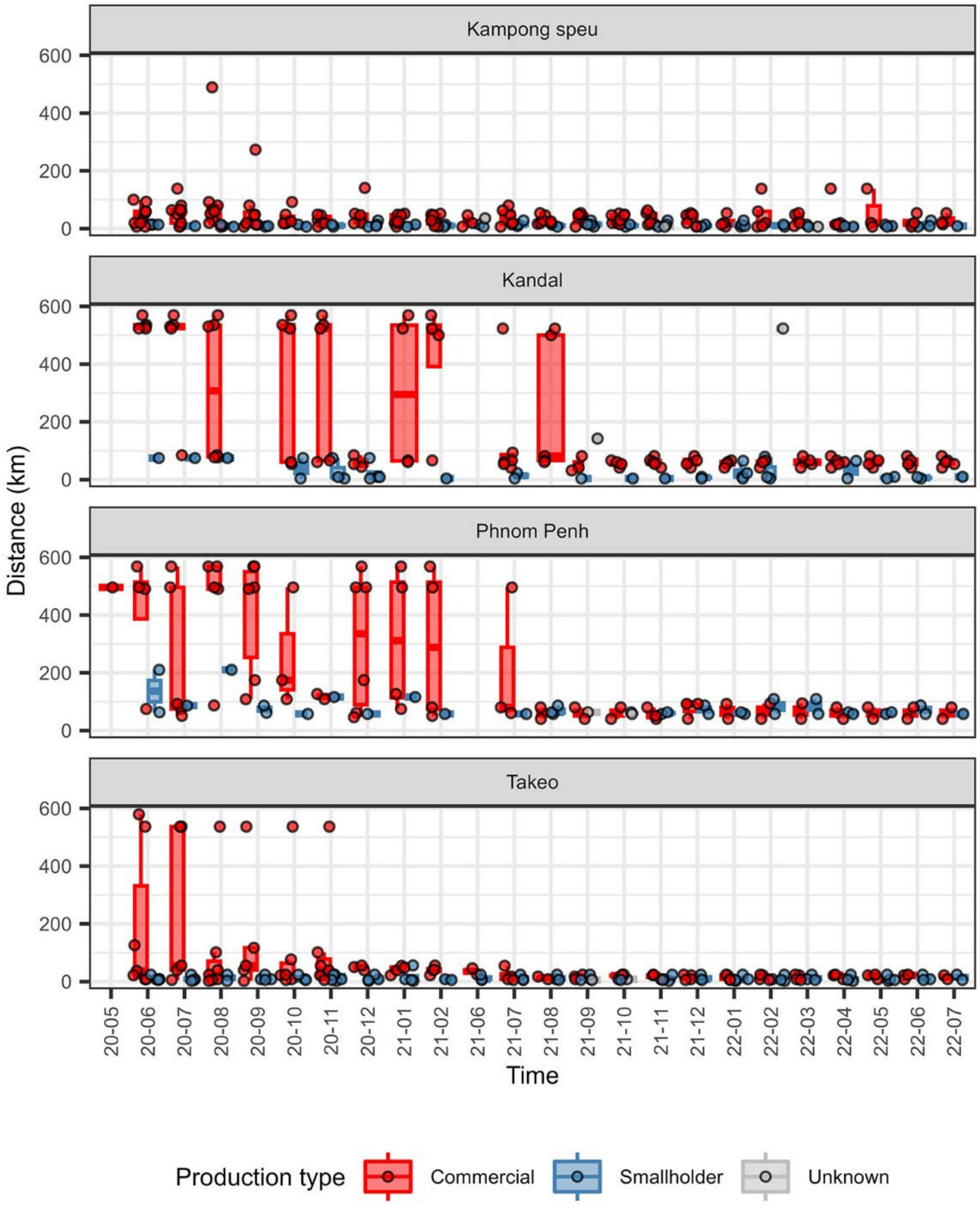

Appendix Figure 3. Transport distances stratified by the province of origin and production type over the sampling period.

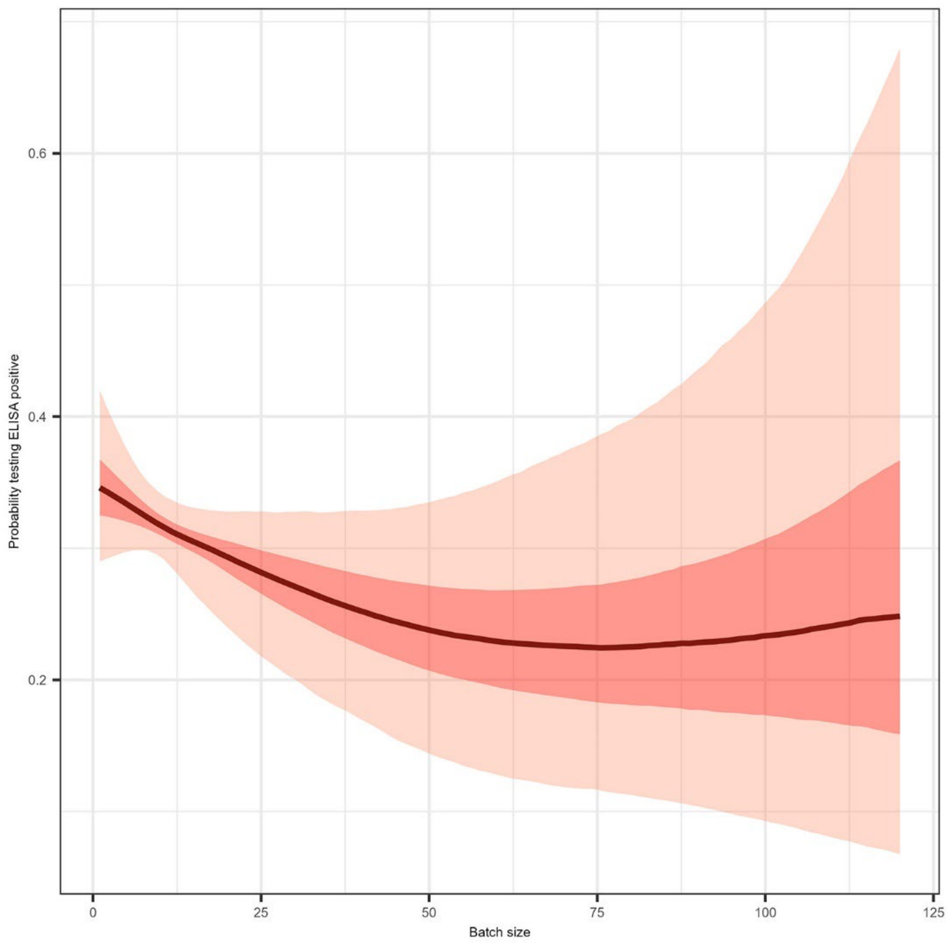

Appendix Figure 4. Estimated effects of batch size on the probability of testing positive for ELISA. Black lines show the posterior coefficient using a generalised additive model. Red areas indicate 50% and orange 95% credible intervals.

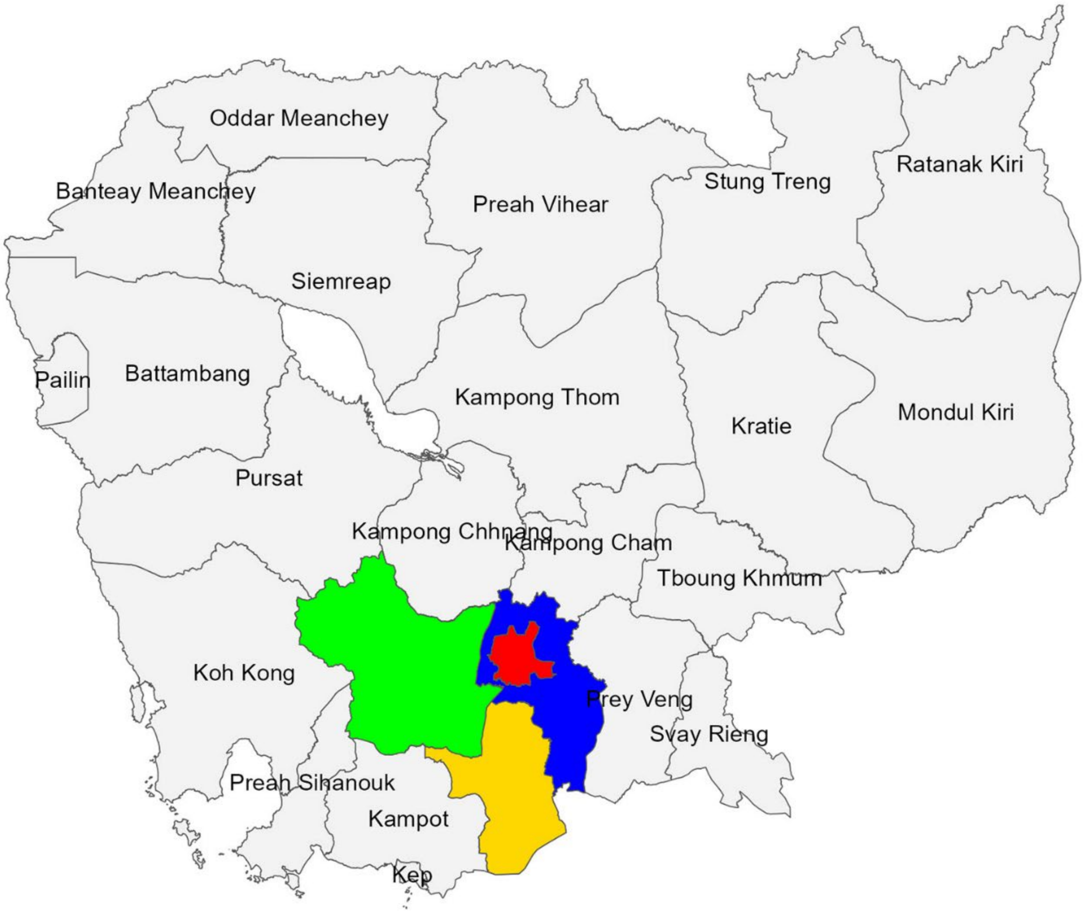

Appendix Figure 5. Map of Cambodia with 4 included provinces. Areas are labeled red, Phnom Penh; blue, Kandal; yellow, Takéo; green, Kampong Speu.

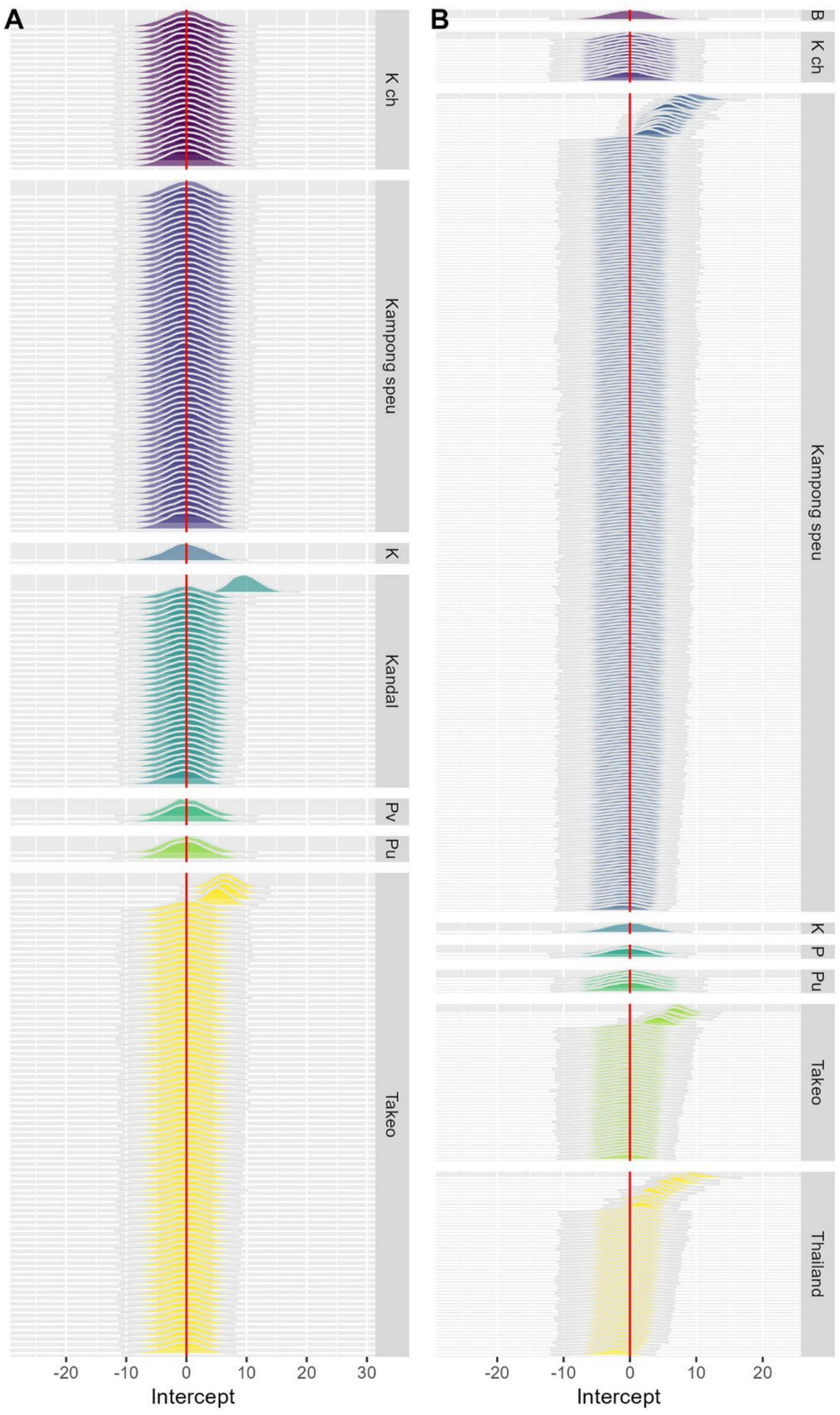

Appendix Figure 6. Distribution of random effect for batches from (A) smallholders and (B) commercial farms on the PCR outcome, stratified by origin province. K ch, Kampong Chhnang; K, Kampot; Pv, Prey Veng; Pu, Pursut; B, Battambang; p, Phnom Penh.

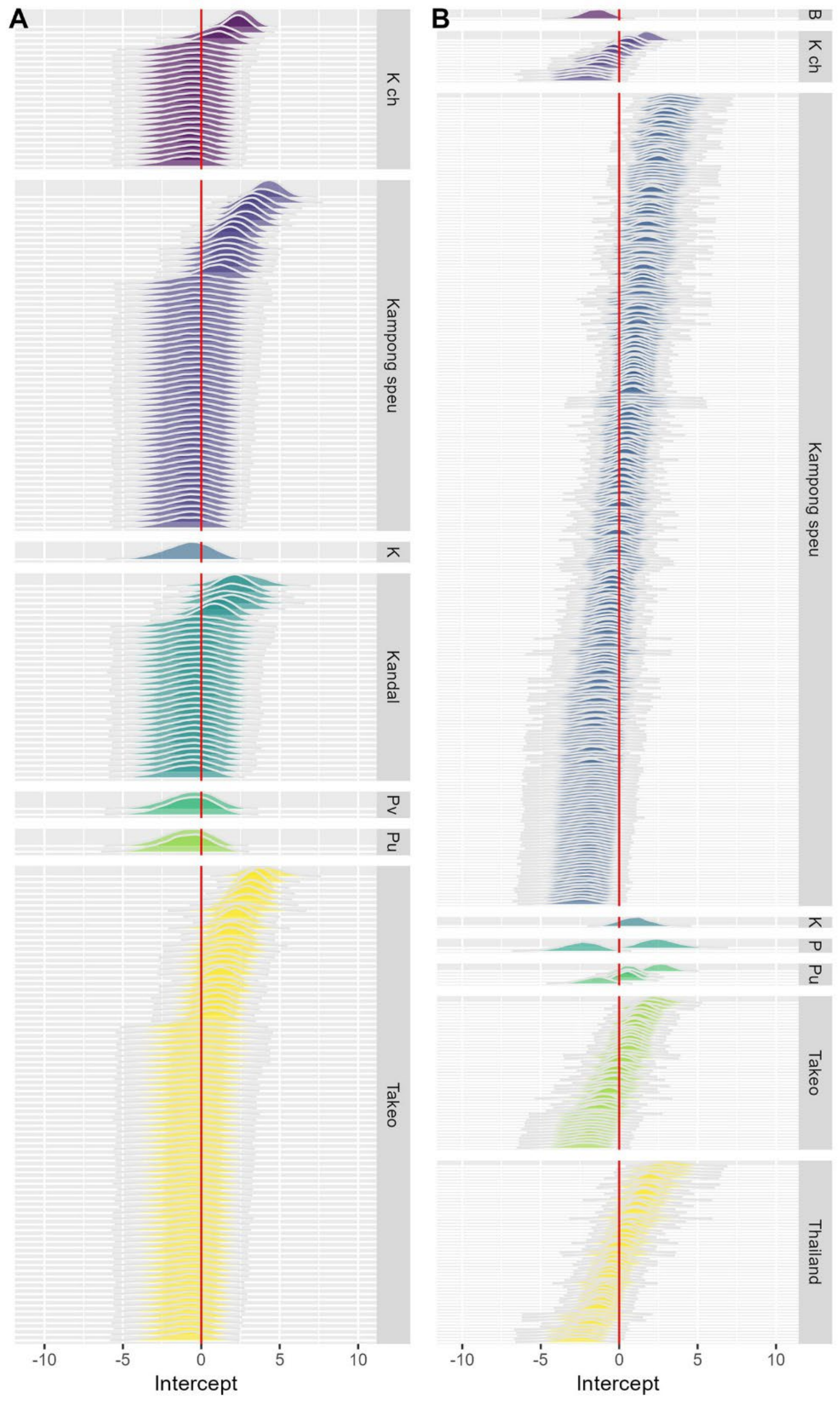

Appendix Figure 7. Distribution of random effect for batches from (A) smallholders and (B) commercial farms on the ELISA outcome, stratified by origin province. K ch, Kampong Chhnang; K, Kampot; Pv, Prey Veng; Pu, Pursut; B, Battambang; p, Phnom Penh.

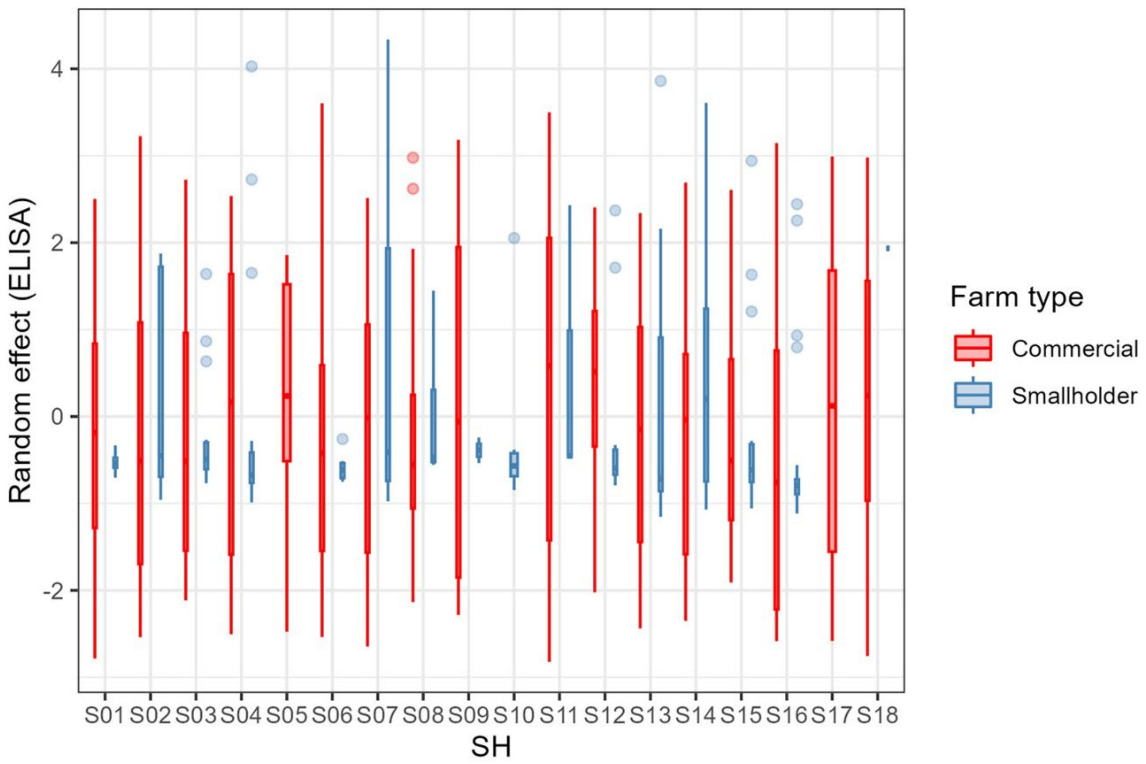

Appendix Figure 8. Distribution of random effect for the ELISA outcome for batches from commercial farms and smallholders across SH (slaughterhouses).

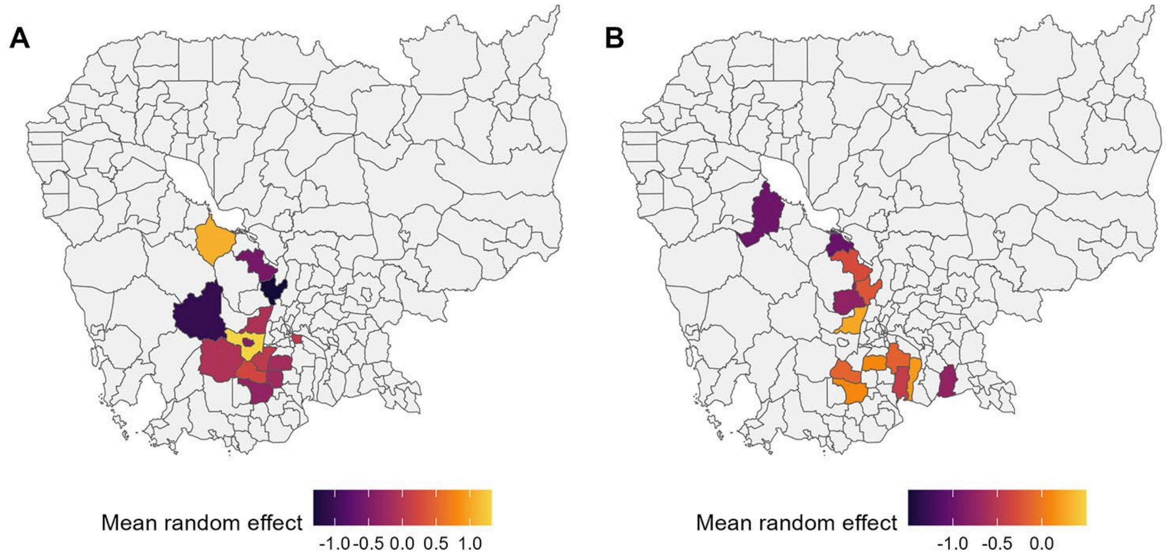

Appendix Figure 9. Distribution of the mean random effect of the ELISA outcome for batches from (A) commercial farms and (B) smallholders across districts.

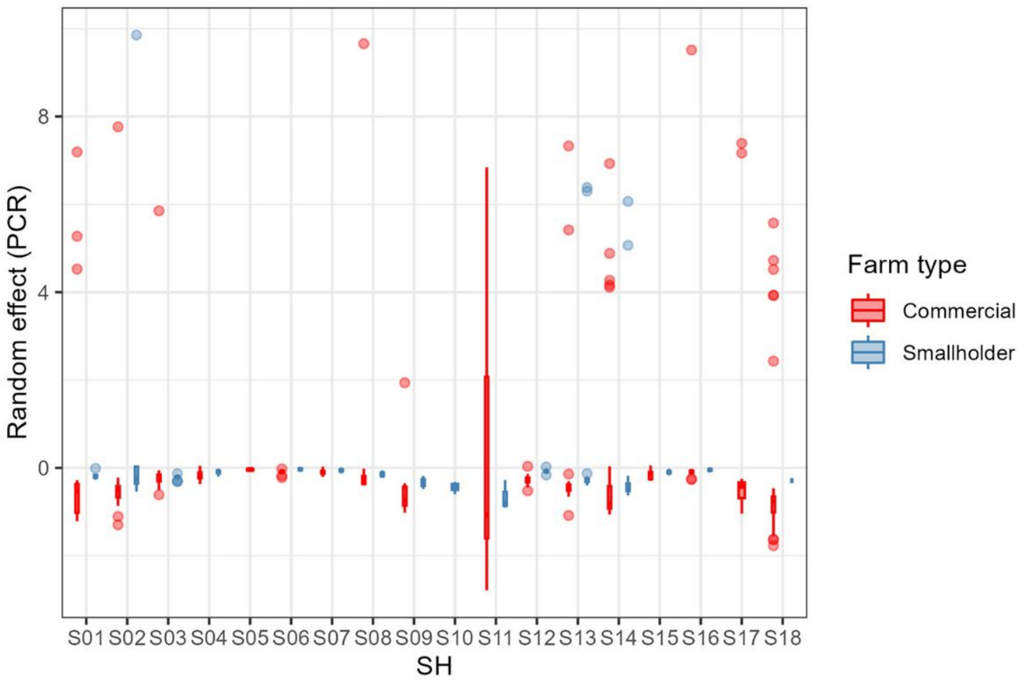

Appendix Figure 10. Random effect distributions of the PCR outcome for slaughterhouses (SH) stratified by farm type.

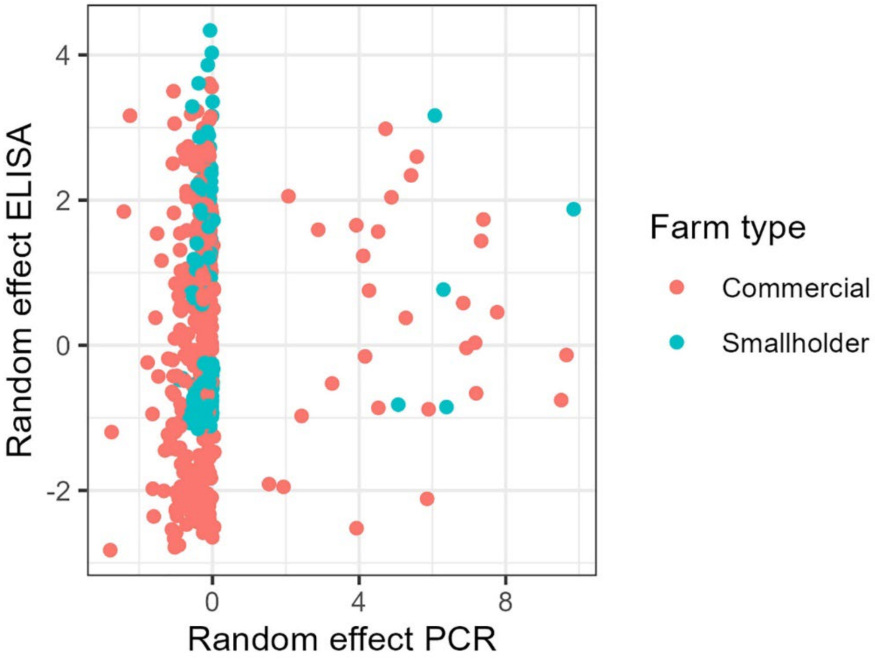

Appendix Figure 11. Scatter plot showing the random effect for PCR (x-axis) and ELISA (y-axis), stratified by farm type.
